# Design, synthesis, and anticonvulsant effects evaluation of nonimidazole histamine H_3_ receptor antagonists/inverse agonists containing triazole moiety

**DOI:** 10.1080/14756366.2020.1774573

**Published:** 2020-06-12

**Authors:** Mingxia Song, Rui Yan, Yanhui Zhang, Dongfu Guo, Naiming Zhou, XianQing Deng

**Affiliations:** a Medical College, Jinggangshan University, Ji’an, China; b College of Life Sciences, Zhejiang University, Hangzhou, China

**Keywords:** Epilepsy, anticonvulsant, H_3_ receptor antagonists/inverse agonists, hybrid, 1,2,4-triazole

## Abstract

Histamine H_3_ receptors (H_3_R) antagonists/inverse agonists are becoming a promising therapeutic approach for epilepsy. In this article, novel nonimidazole H_3_R antagonists/inverse agonists have been designed and synthesised *via* hybriding the H_3_R pharmacophore (aliphatic amine with propyloxy chain) with the 1,2,4-triazole moiety as anticonvulsant drugs. The majority of antagonists/inverse agonists prepared here exerted moderate to robust activities in cAMP-response element (CRE) luciferase screening assay. 1-(3-(4-(3-Phenyl-4*H*-1,2,4-triazol-4-yl)phenoxy)propyl)piperidine (**3l**) and 1-(3-(4-(3-(4-chlorophenyl)-4*H*-1,2,4-triazol-4-yl)phenoxy)propyl)piperidine (**3m**) displayed the highest H_3_R antagonistic activities, with IC_50_ values of 7.81 and 5.92 nM, respectively. Meanwhile, the compounds with higher H_3_R antagonistic activities exhibited protection for mice in maximal electroshock seizure (MES)-induced convulsant model. Moreover, the protection of **3m** against the MES induced seizures was fully abrogated when mice were co-treated with RAMH, a CNS-penetrant H_3_R agonist, which suggested that the potential therapeutic effect of **3m** was through H_3_R. These results indicate that the attempt to find new anticonvulsant among H_3_R antagonists/inverse agonists is practicable.

## 1. Introduction

Epilepsy, a very common neurologic disorder, affects about around 1% of world population^1^^,^^2^. Presently, antiepileptic drugs (AEDs) are the main strategy of therapy. However, the AEDs available in the clinic such as phenytoin, carbamazepine, sodium valproate, topiramate, and oxcarbamazepine are only effective in approximately 70% of the patients with epilepsy. Moreover, their use is long-term and often accompanied with severely side effects, including naupathia, headache, and ataxia, even threaten the life of patients^3–5^. Investigations for more effective and safer AEDs are still a formidable and urgent task for medicinal chemists.

The role of central histaminergic system being concerned in epilepsy have been demonstrated in many experimental and epidemiological studies, in which histamine regulated seizure susceptibility as an anticonvulsant neurotransmitter^6–8^. For example, H_1_-antagonists such as pyrilamine, ketotifen that decrease brain histamine levels increased the duration of convulsive phase in electrically-induced convulsions model^9^. Histidine, as the precursor of histamine, showed protection against chemically-induced convulsions in rats, *via* activating the histamine H_1_ receptors^10^.

Histamine H_3_ receptors (H_3_R) as a G-protein coupled receptor (GPCR) binding to histamine like other histamine receptors, is expressed mainly in the central nervous system, where it acts as an auto-receptor in histaminergic neurons, and negatively regulates the synthesis and release of histamine^11^. What is more, as a inhibitory heteroreceptor, H_3_R also regulates the release of other neurotransmitters including dopamine, acetylcholine, serotonin, norepinephrine, γ-aminobutyric acid, and glutamate. These neurotransmitters, especially γ-aminobutyric acid and glutamate, are related to epilepsy inextricably^12^^,^^13^. Therefore, more attention has been focussed on H_3_R as an attractive therapeutic target for epilepsy treatment^14^.

A large number of experimental studies involved in acute and chronic models of epilepsy confirmed the anticonvulsive potential of H_3_R antagonists/inverse agonists. They showed the protection against experimental seizures by feedback increase of histamine release and binding with H_1_ receptors^15^^,^^16^. Besides, other mechanisms might be involved in their anticonvulsive action, such as facilitating of GABA release^17–19^, increasing histidine decarboxylase (HDC) activity^20^^,^^21^ and synergism with AEDs^17^^,^^18^^,^^22^.

**Figure 1.**
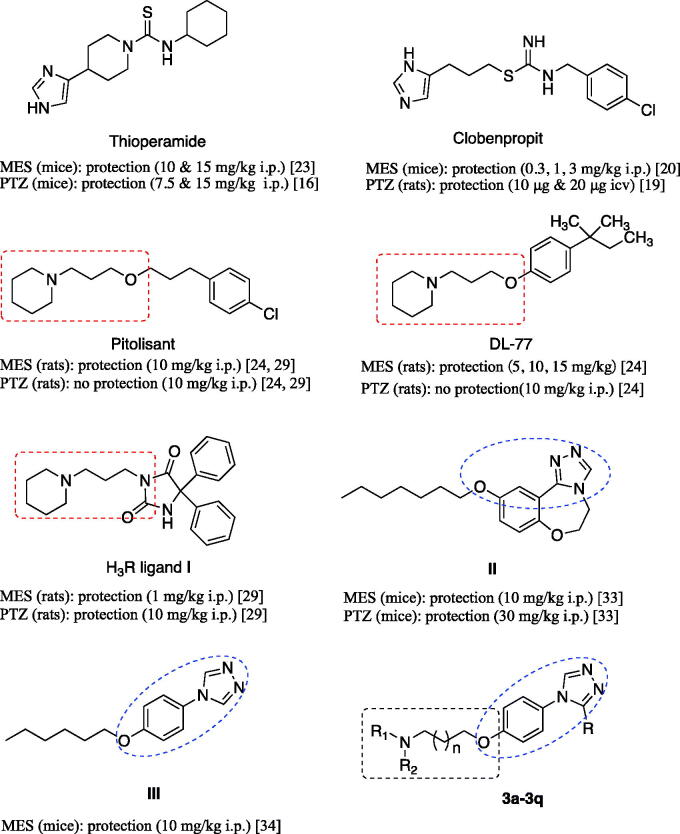
Structures of histamine H_3_ receptor ligands with anticonvulsant activity, triazole derivatives with anticonvulsant activity and target compounds **3a-3q** designed.

Early, anticonvulsant activity of some imidazole H_3_R antagonists such as thioperamide and clobenpropit was confirmed in models of epilepsy (Figure 1)^16^^,^^19^^,^^20^^,^^23^. Recently, a large number of non-imidazole H_3_R antagonists such as DL77 (Figure 1) prepared by a group/team of Kiec-Kononowicz exhibited excellent anticonvulsant activity in the electrically-induced seizures model and subcutaneously pentylenetetrazole (PTZ)-induced seizure model at dose-dependent, and the therapeutic action was proved through H_3_R^24–28^. Sadek et al. synthesised some histamine H_3_R ligands (Figure 1, I) incorporating different antiepileptic structural motifs to investigate if the H_3_R pharmacophore could be combined to some antiepileptic molecules, and give some new anticonvulsants by the multiple-target approaches. The results were encouraging, which indicated that the H_3_R pharmacophore successfully combined to the antiepileptic molecules, maintaining the H_3_R affinity and anticonvulsant activity, although the anticonvulsant activity decreased compared to the prototypal antiepileptic molecules (Figure 1)^29^^,^^30^.

Pitolisant (PIT), a H_3_R antagonist/inverse agonist, has been subjected into clinical Phase III for the treatment of epilepsy^31^. When used alone or in combination with other AEDs in the human photosensitivity model at dose ranges of 30–60 mg, PIT showed a favourable EEG profile in a dose-dependent manner^32^.

Supported by the above results, in this work, we designed and synthesised some novel H_3_R antagonists/inverse agonists by hybriding the H_3_R pharmacophore (aliphatic amine with propyloxy chain) with the 1,2,4-triazole, the latter have been identified as an important and effective anticonvulsive fragment in recent years (Figure 1, II and III)^33–37^. According to Quan’s reports, the 1,2,4-triazole derivatives were likely to have several mechanisms of action such as inhibiting voltage-gated sodium ions channel and modulating GABAergic activity^38–40^. And a group of Plech illustrated the anticonvulsive effects of 4-alkyl-5-aryl-1,2,4-triazole-3-thione derivatives and suggested that the influence on the voltage-gated Na^+^ channels was involved in them at least^41^^,^^42^. Therefore, in this work, our strategy was to design molecules combining pharmacophores of H_3_R antagonists and another anticonvulsant active pharmacophore (e.g. 1,2,4-triazole moiety) into one skeleton, and then produced a synergism for anticonvulsant active.

## 2. Results and discussion

### 2.1. Chemistry

**Scheme 1.**
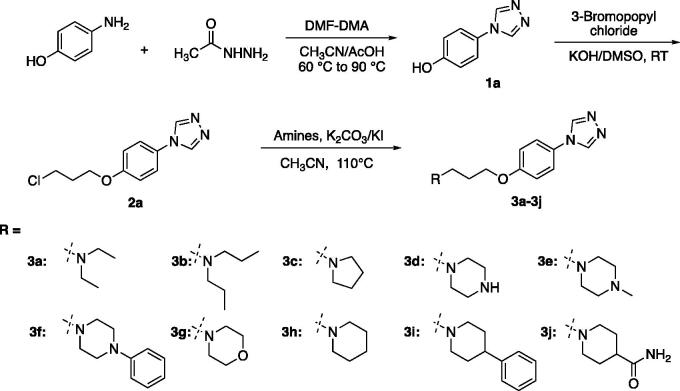
The synthesis route and conditions for the preparation of compounds **3a-3j.**

**Scheme 2.**
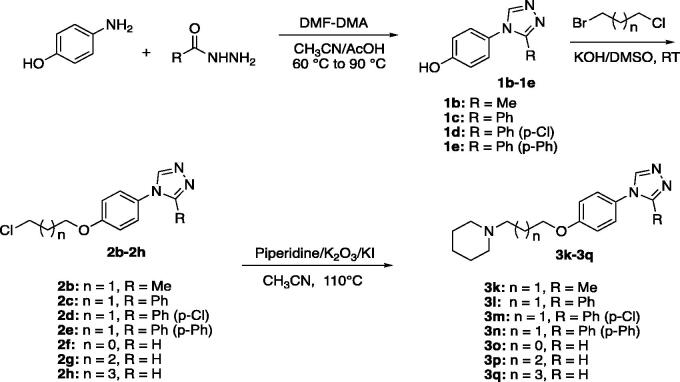
The synthesis route and conditions for the preparation of compounds **3k-3q.**

According to Schemes 1 and 2, the target compounds (**3a-3q**) were synthesised smoothly. In brief, formyl hydrazine reacted with 4-aminophenol in dimethoxyl-*N,N*-dimethyl formamide (DMF-DMA) to give the 4-(4*H*-1,2,4-triazol-4-yl)phenol (**1a**). Compound **1a** underwent a nucleophilic substitution with 1-bromo-3-chloropropane to get 4-(4-(3-chloropropoxy)phenyl)-4*H*-1,2,4-triazole (**2a**). The reaction was conducted in the presence of potassium hydroxide in dimethyl sulfoxide (DMSO) at room temperature to ensure the formation of single-substituted derivatives. Finally, proper amines reacted with compound **2a** in the presence of K_2_CO_3_ and KI in the solvent of CH_3_CN to give the desired compounds **3a-3j**. To enrich the structure–activity relationship, we also prepared the derivatives of **3h** *via* introducing the substituents at the triazole ring and adjusting the length of the link. The reaction conditions used to prepare these compounds (**3k-3q**) were the same as above. Compounds (**3a**, **3b**, **3c**, **3d**, **3e**, **3 g**, **3k**, **3o**, and **3p**) obtained as the form of oil were transformed to hydrochlorate. Their structures were characterised and confirmed by^1^H-NMR, ^13 ^C-NMR, and HR-MS.

### 2.2. Biological activities

#### 2.2.1. Evaluation of H_3_R antagonistic activity

cAMP-response element (CRE) reporter gene assay has been extensively used to evaluate the efficacy of GPCR antagonists or agonists. In this work, the H_3_R antagonistic activities of the prepared 3-(4-(4*H*-1,2,4-triazol-4-yl)phenoxy)-propylamine derivatives have been screened by CRE-driven luciferase assay, in which the HEK-293 cells expressing the human H_3_R and a reporter gene consisting of the firefly luciferase coding region were used^43^^,^^44^. Ciproxifan (CXP) and Pitolisant (PIT) were employed as the positive controls. Initially, compounds and positive controls were tested at two concentrations (100 nM and 1 µM) to obtain the preliminary investigation of their H_3_R antagonistic activities. In the assays, the antagonistic activities was positively correlated with the rise of the fluorescence value and indicated by the % antagonism. For the prominent compounds IC_50_ values were determined at additional assays.

As seen in Table 1, majority of the synthesised compounds displayed gentle to robust H_3_R antagonistic activities and eight of them exhibited micromolar inhibitory activity. The antagonistic activities of all compounds depended on the concentration treated. It is worth mentioning that compounds **3l** (IC_50_ = 7.81 nM) and **3m** (IC_50_ = 5.92 nM) displayed the most potent H_3_R antagonistic activities, with the much stronger potency than that of CXP (IC_50_ = 0.082 µM) and PIT (IC_50_ = 0.5 µM) in the CRE reporter gene assay.

**Table 1.** H_3_R antagonistic activity of compounds **3a-3q**.

Compounds	*R*	*n*	% Antagonism^a^		H_3_R antagonistic activity (IC_50_, μM)
			100 nM	1 μM	
			
**3a**			49.85 ± 9.73	223.76 ± 7.56	2.99
**3b**			13.24 ± 4.83	15.02 ± 4.03	NT^b^
**3c**			32.23 ± 5.69	143.98 ± 16.16	0.553
**3d**			11.33 ± 5.86	79.4 ± 4.50	NT
**3e**			9.86 ± 0.87	21.78 ± 3.04	NT
**3f**			9.96 ± 10.9	24.41 ± 6.30	NT
**3g**			−0.76 ± 4.87	20.24 ± 7.56	NT
**3h**			10.56 ± 7.94	239.79 ± 3.17	0.127
**3i**			5.19 ± 2.06	16.45 ± 1.90	NT
**3j**			−4.2 ± 3.11	6.12 ± 2.05	NT
			
**3k**	Me	1	133.91 ± 8.91	183.54 ± 14.41	0.021
**3l**	C_6_H_5_	1	148.62 ± 4.43	169.04 ± 15.04	0.00781
**3m**	C_6_H_4_(p-Cl)	1	201.12 ± 12.63	203.92 ± 8.44	0.00592
**3n**	Biphenyl	1	1.03 ± 7.79	3.36 ± 1.27	NT
**3o**	H	0	45.04 ± 3.61	94.20 ± 2.60	0.25
**3p**	H	2	25.00 ± 3.95	90.52 ± 5.71	3.44
**3q**	H	3	1.88 ± 12.38	2.39 ± 13.85	NT
**CPX^c^**			33.96 ± 8.59	63.05 ± 9.33	0.082
**PIT^d^**			54.16 ± 7.33	246.29 ± 21.48	0.51

^a^% Antagonism, value represented as mean ± standard deviation of three independent experiments.

^b^NT, IC_50_ was not tested.

^c^CPX, an antagonist of H_3_R ciproxifan.

^d^PIT, an antagonist/inverse agonist of H_3_R pitolisant.

**Figure 2.**
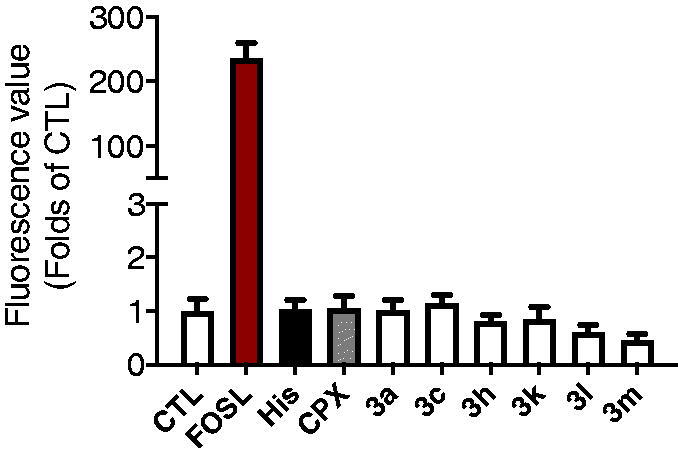
Effects of compounds **3a**, **3c**, **3h**, **3k**, **3l**, and **3m** on the level of intracellular cAMP administrated alone.

Surprisingly, antagonism percent of some compounds as well PIT were above 100%. It is well known that H_3_R is a GPCR coupled with Gαi. When the ligand (histamine) binds to H_3_R, the dissociated Gαi inhibits the activity of adenylate cyclase (AC) and down-regulates the level of intracellular cAMP. In this CREs driven luciferase assay, when cells were pre-treated by H_3_R antagonists, the downregulation of cAMP would be inhibited, and the level of intracellular cAMP would regain to the initial level. However, in some cases, the cAMP levels raised above the initial level, giving the % antagonism greater than 100%. Our first speculation was that these compounds might stimulate the AC directly and up-regulate the level of cAMP. However, an additional assay indicated that these compounds didn’t have effects on the level of cAMP when pre-treated alone. As shown in Figure 2, forskolin (2 µM) treated group gave more than 200 times rise for the cAMP level when compared to the control group. While Histamine, ciproxifan, and compounds **3a**, **3c**, **3h**, **3k**, **3l**, and **3m** have no significant effects on the level of cAMP when carried out comparisons by ANOVA followed by Dunnett’s test.

**Figure 3.**
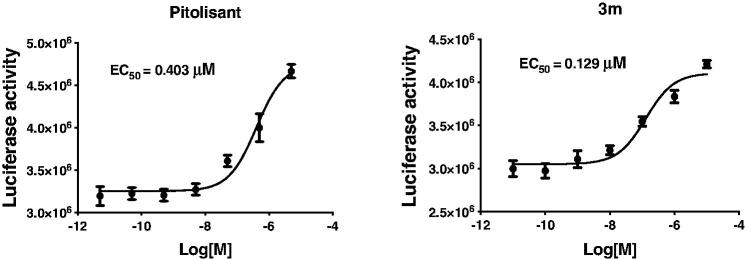
The H_3_R inverse agonistic activity (EC_50_, μM) of Pitolisant and compound **3m**.

Another explanation is that these compounds may be inverse agonists when binding to H_3_R, which not only antagonise the function of histamine, but also give the inverse agonistic performance. Actually, PIT is a well-known H_3_R antagonist and reverse agonist. So, the above results make sense. Based on the above, we further assessed the H_3_R inverse agonist activity of compound **3m** and PIT by using CRE-luciferase assay. In this experiment, transfected HEK-293 cells were stimulated with 10 µM forskolin or 10 µM forskolin plus different concentrations of compound **3m**. The raise of luciferase activity after adding compound represented the inverse agonistic activity. The EC_50_ was calculated by seven concentrations. Cytotoxicity appeared at 100 µM. As shown in Figure 3, PIT and compound **3m** showed effective H_3_R inverse agonistic activity with an EC_50_ value of 403 and 129 nM, respectively.

Simple structure–activity relationships (SARs) could be obtained from Table 1. In the series of **3a-3j**, the different tertiary amines significantly influenced the H_3_R antagonistic activities. The *N*-ethyl derivative **3a** showed an IC_50_ of 2.9 µM, while the activity declined sharply for the *N*-propyl derivative **3b**. Interestingly, compounds containing piperazine or morpholine (**3d-3g**) exhibited weaker activities than those with piperidine or pyrrolidine (**3c** and **3h**). This probably attributed to the increase of the molecular polarity. The introduction of phenyl or amide group on the piperidine ring of compound **3h**, gave the compounds **3i** and **3j**, which also decreased the H_3_R antagonistic activities when compared to compound **3h**. Based on the facts above, it could be concluded that the *N,N*-diethyl group, pyrrolidine and piperidine were more of benefit to the H_3_R antagonistic activities of the 3-(4-(4*H*-1,2,4-triazol-4-yl)phenoxy)-propylamine skeleton, and piperidine derivative (**3h**) was the best one with the IC_50_ of 0.127 µM.

To enrich the structure–activity relationships, we prepared the derivatives of **3h** *via* introducing the substituents at the triazole ring and adjusting the length of the link.

Compounds **3k**, **3l**, **3m**, and **3n** were substituted on 3-position of 1,2,4-triazole ring with methyl, phenyl, *para-*chlorophenyl, and biphenyl, respectively. Encouragingly, the introduction of methyl, phenyl, and *para*-chlorophenyl groups significantly increased the H_3_R antagonistic activities, giving the two prominent compounds **3l** and **3m** with nanomolar IC_50_ values. While the biphenyl substituted compound 3**n** showed weaker activity when compared to **3h**. Replacing the three-carbon link in the compound **3h** with two-carbon, four-carbon and five-carbon links, gave the compounds **3o**, **3p**, and **3q**, respectively. It could be seen that the length of the link had a direct impact on H_3_ receptor antagonistic activities of the 3-(4-(4*H*-1,2,4-triazol-4-yl)phenoxy)-propylamine derivatives. The activity order of the link length of carbon was 3 > 2 > 4 ≫ 5.

**Figure 4.**
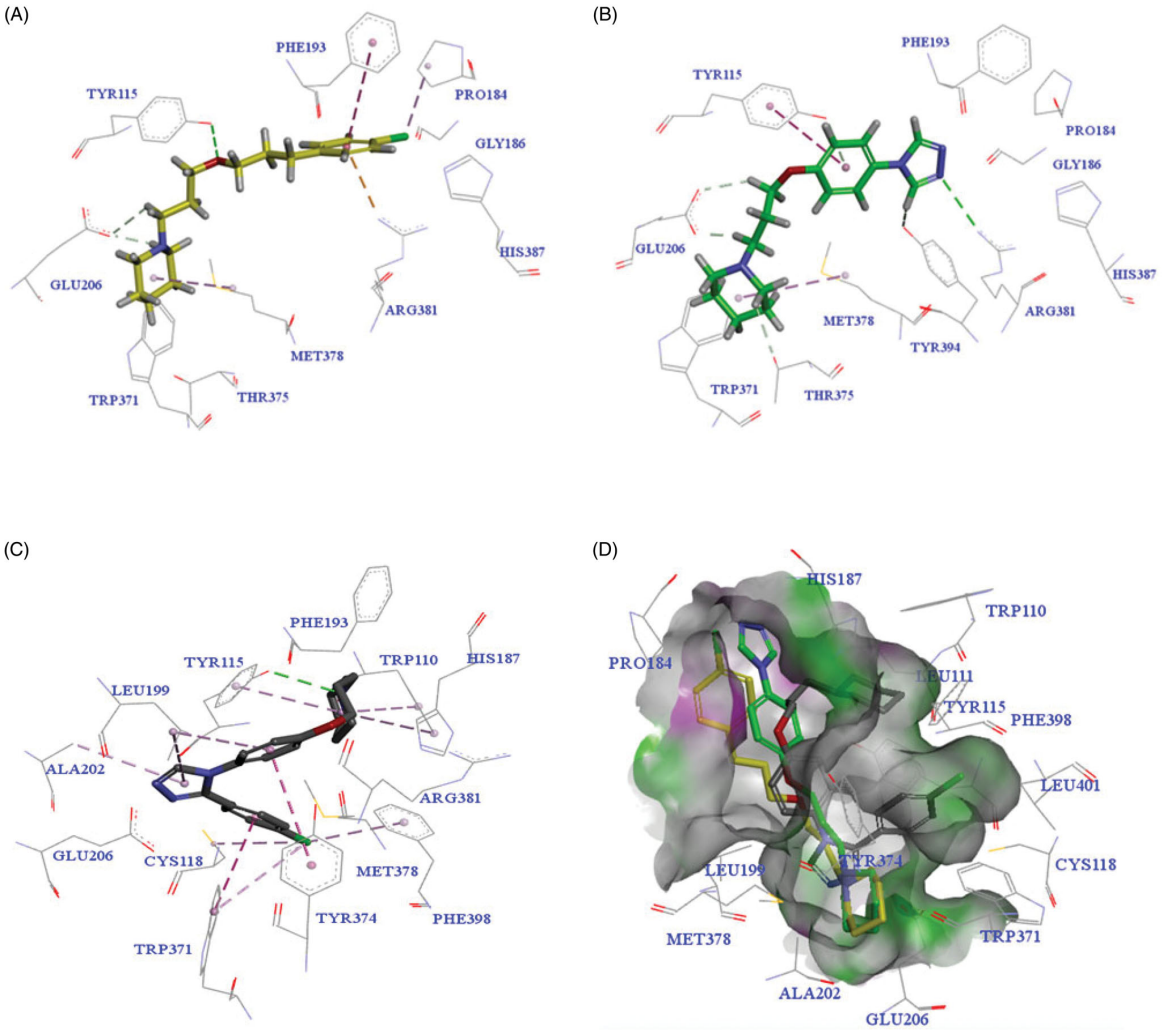
The predicted configurations for **PIT** (A), **3h** (B) and **3m** (C) binding with H_3_R, and their overlying pattern (D).

To investigate the molecular determinants that manage the antagonistic activities of the tested compounds, molecular docking studies of PIT, **3h**, and **3m** with the H_3_R homology model were carried out. The homology model was constructed from the crystal structure of the H_1_ receptor (PDB ID: 3RZE)^45^. The docking results are shown in Figure 4.

As shown in Figure 4(A), PIT bound to H_3_R through two critical H-bond interactions with Tyr115 and Glu206, and other interactions with amino acid residues Arg381, Phe193, Met378 and so on. Figure 4(B) revealed that compound **3h** had a similar binding pattern to PIT, interacting with the same amino acid residues Glu206, Tyr115, Arg381, and Met378. Surprisingly, the compound **3m** with the highest H_3_R antagonistic activity showed a different binding pattern to PIT (as seen in Figure 4(C)). The overlying pattern of PIT, **3h**, and **3m** was shown in Figure 4(D). The piperidine group of **3m** was not involved in the formation of the salt bridge or hydrogen-bond interactions with Glu206, which was generally considered as the critical residue of H_3_R^46^^,^^47^. The unexpected binding pattern of **3m** might be due to the phenyl group on the triazole ring, which did not fit into the hydrophobic cavity in TMs 3-5-6 region of H_3_R, even though the compound **3m** showed a forceful binding with H_3_R *via* another mode. The triazole nitrogen established an ionic bond with Glu206, and a hydrogen bond was observed between piperidine nitrogen and Tyr115. π-π shaped, and alkyl interactions with Trp371, Tyr343, Arg381, His187, Leu199, and ALA202 were observed to support the forceful binding with H_3_R.

#### 2.2.2. Anticonvulsant activity evaluation

To investigate the anticonvulsive effects, all the target compounds (**3a-3q**) were screened in the MES-induced and PTZ-induced convulsion models in mice. Compounds were administered intraperitoneally (*i.p.*) to mice at dosage of 10 mg/kg in the both models. PIT and valproic sodium (VPA) were used as positive controls in the tests.

##### 2.2.2.1. Protective effects of H_3_R antagonists/inverse agonists 3a-3q on MES-induced convulsions

**Figure 5.**
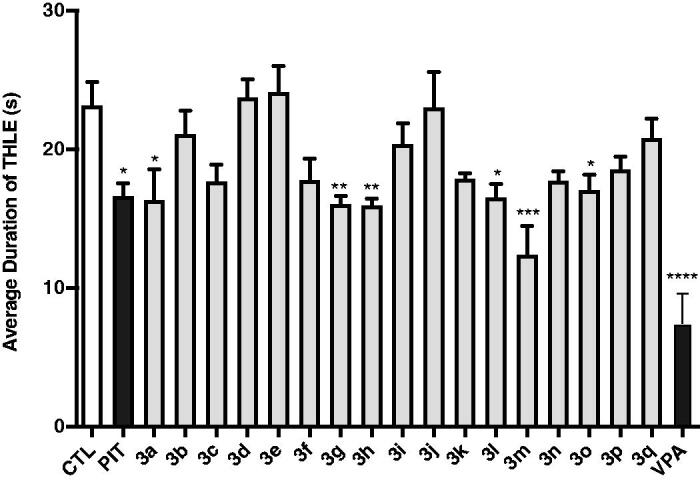
Effects of H_3_R antagonists/inverse agonists **3a-3q** (10 mg/kg, *i.p.*), PIT (10 mg/kg, *i.p.*) and anticonvulsant drug VPA (300 mg/kg, *i.p.*) against MES-induced convulsions. Protection for mice was defined as the reduction or abolition of the tonic hind limb extension (THLE) in MES model. Results are showed as mean ± SEM with seven animals in each group. Values are considered significant at *p < 0.05, **p < 0.01, ***p < 0.001, ****p < 0.0001 as compared to saline-treated group.

Protection for the mice was defined as the reduction or abolition of the tonic hind limb extension (THLE) in the MES model in mice. As seen in Figure 5, compounds **3a**, **3 g**, **3h**, **3l**, **3m**, and **3o** showed moderate protection for the electro-stimulated mice with significant difference from that of the control group (*p* < 0.05, *p* < 0.01, or *p* < 0.001). Mice pre-treated with PIT (10 mg/kg, *i.p.*) and VPA (300 mg/kg, *i.p.*) were moderately or potently protected, respectively. Generally, the anticonvulsant activities of these compounds in MES model correlated directly to their H_3_R antagonistic activities. For example, the antiepileptic activity obtained of compound **3m** was the highest, and *in vitro* H_3_R antagonistic activity measured for **3m** with IC_50_ of 5.92 nM was also the highest. Compounds **3a**, **3h**, **3l**, and **3o**, showing anticonvulsant activity in the MES model, also showed good H_3_R antagonistic activities. Compound **3c**, **3k**, and **3p** reduced the average duration of THLE, although they did not achieve a significant difference from the control group.

##### 2.2.2.2. Protective effects of H_3_R antagonists/inverse agonists 3a-3q on PTZ-induced convulsions

**Figure 6.**
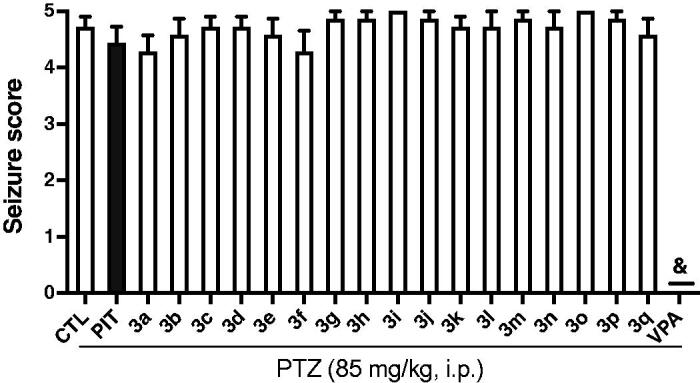
Effects of compounds **3a-3q** (10 mg/kg, *i.p.*), and reference drug PIT (10 mg/kg, *i.p.*) and VPA (300 mg/kg, *i.p.*) against PTZ-induced convulsions. Results are showed as mean ± SEM of seven mice in each group. & represent full protection.

Some experiments indicated that H_3_R antagonists/inverse agonists could protect animals in PTZ-induced convulsions model^26^^,^^29^. So the compounds **3a-3q**, PIT, and VPA were also screened in the PTZ model in mice. Unfortunately, all compounds tested at the dose of 10 mg/kg (*i.p*.) did not show any protection against the seizures induced by PTZ. PIT also failed to protect the PTZ-treated mice as well at the same conditions. By contrast, anticonvulsant agent VPA showed full protection against the PTZ-induced convulsions (Figure 6).

##### 2.2.2.3. Effects of compound 3m on MES-induced convulsions in dose dependent manner

**Figure 7.**
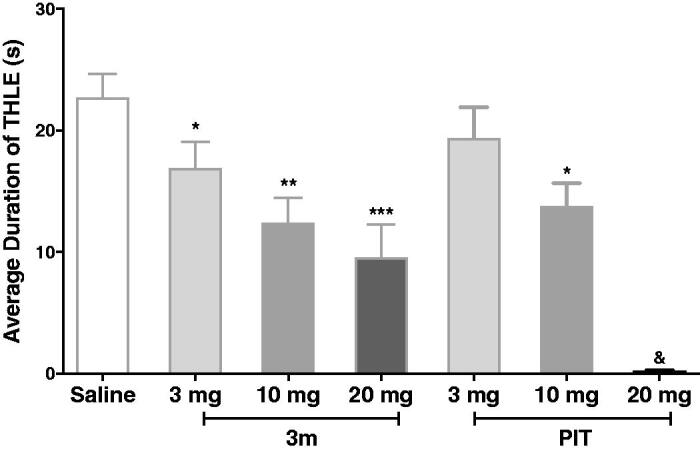
Protective effects of compound **3m** and reference drug PIT against MES-induced convulsions in different doses. Protection in the test was defined as the reduction or abolition of the THLE in mice. Results were showed as mean ± SEM with seven animals in each group. Values are considered significant at *p < 0.05, **p < 0.01, ***p < 0.001 when compared to saline-treated group. ^&^PIT, at 20 mg/kg dose, fully abrogate the THLE for all the tested mice.

In a further experiment, compound **3m**, as the most active one in the MES-induced seizure model, was chosen to verify its protective effect in different doses. Encouragingly, the **3m**-provided protections were observed and were dose dependent. The standard antagonist PIT also displayed anticonvulsive activity dose-dependently at the same condition. Notably, when pre-treated with 20 mg/kg dose, PIT could fully abrogate the tonic hind limb extension induced by electro-stimulation, showing its potential anticonvulsant activity (Figure 7). To exclude the possibility that the anticonvulsant activity of **3m** was connected with sedative effect, we carried out a rotarod test for **3m**. The result showed that compound **3m** had no neurotoxicity at the maximum dose of 10 and 20 mg/kg (the details could be seen in Support Table 1).

##### 2.2.2.4. Effects of RAMH pre-treatment on the compound 3m-provided protection in MES-induced seizure model

**Figure 8.**
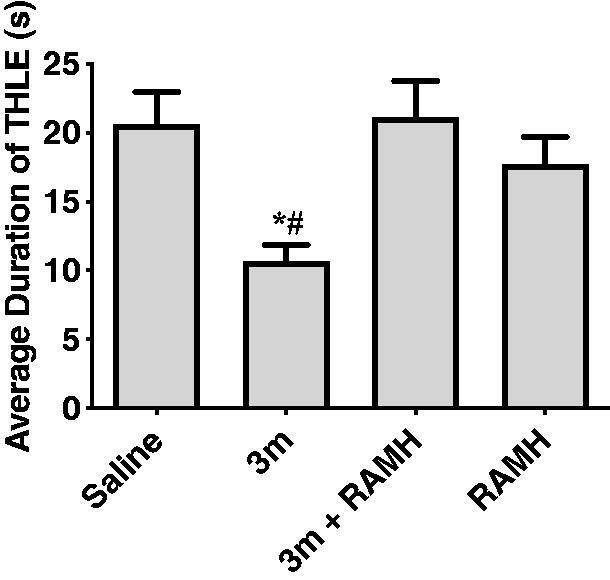
Protective effects of compound **3m** (10 mg/kg, *i.p.*) against MES-induced convulsions when pre-treatment of RAMH (10 mg/kg, *i.p.*). Protection in the test was defined as the reduction or abolition of the THLE in mice. Results are showed as mean ± SEM with seven animals in each group. Values are considered significant at *p < 0.01 as compared to saline-treated group, and ^#^*p* < 0.01 as compared to 3**m** + RAMH treated group.

To investigate the correlation between the anticonvulsant activity and H_3_R antagonistic activity of compound **3m**, the protection provided by compound **3m** against MES-induced seizure was reassessed after the administration of RAMH (10 mg/kg, *i.p.*), a CNS penetrant histamine H_3_R agonist. The results indicated that when co-administration with RAMH, compound **3m** lost its original protective effect (Figure 8). Administration of RAMH alone also did not affect the duration of THLE of mice with *p* > 0.05 for saline *versus* RAMH. The above findings suggested that H_3_R antagonism was the main contributor for the anticonvulsant activity of compound **3m** in MES model. When the H_3_R was blocked by H_3_R antagonist **3m**, histamine or other neurotransmitter such as GABA in the CNS increased, finally leading to anticonvulsive effects.

## 3. Conclusion

To identify novel H_3_R antagonists/inverse agonists with potential anticonvulsant activities, a series of 3-(4-(4*H*-1,2,4-triazol-4-yl)phenoxy)-propylamine derivatives were designed through combining pharmacophore of H_3_R antagonists and another anticonvulsant active pharmacophore (1,2,4-triazole moiety) into one molecule. The majority of those prepared compounds displayed moderate to robust H_3_R antagonistic activities. The SAR analysis revealed that piperidine and triazolephenol linked by three-carbon chain was benefit for the H_3_R antagonistic activity, and substitution by aromatic nucleus on the 3-position of 1,2,4-triazole further increased the H_3_R antagonistic activities. The most potent H_3_R antagonists/inverse agonists **3l** and **3m** exhibited nanomolar H_3_R antagonistic activities with IC_50_ of 7.81 nM and 5.92 nM, respectively. Molecular docking analysis demonstrated that **3m** strongly bound to H_3_R *via* interactions with Tyr115, Glu206, Trp371, Tyr343, and so on, although its binding mode was not similar to PIT. The anticonvulsive screens *in vivo* indicated that compounds with higher H_3_R antagonistic activities showed more protection in the MES-induced convulsant model in mice, while no one was observed protective effect in PTZ-induced convulsant model. Moreover, the protection of **3m** in the seizure model was fully abrogated when mice were co-treated with a H_3_R agonist RAMH, which suggested that its potential therapeutic effect was through H_3_R.

## 4. Experimental section

### 4.1. Synthesis

All the chemical solvents and reagents were purchased from supplier and used as received. Unless otherwise specified, reactions were monitored by thin-layer chromatography (TLC). All NMR spectrum was carried out on an AV-300 spectrometer with 300 MHz. High resolution mass spectra were measured on an MALDI-TOF/TOF mass spectrometer.

#### 4.1.1. Synthesis of compounds 1a-1e

Taking compound **1a** as an example: dimethoxyl-*N, N*-dimethyl formamide (DMF-DMA, 1.31 g, 11 mmol) and formyl hydrazine (0.65 g, 11 mmol) were added into a flask containing 30 ml of acetonitrile. The mixture was heated up to 60 °C for 30 min, then 4-aminophenol (0.60 g, 5.5 mmol) and acetic acid (3 mL) were added and heated up to 120 °C for 9 h. The mixture was cooled, filtered and washed by acetonitrile to give the product **1a**. Chemical formula: C_8_H_7_N_3_O (MW = 161.16). m.p. 270–272 °C, yield 73%. ^1^H-NMR (300 MHz, DMSO-d_6_): *δ* 6.90 (d, 2H, *J* = 8.9 Hz, Ph-H), 7.46 (d, 2H, *J* = 8.9 Hz, Ph-H), 8.94 (s, 2H, N = CH), 9.88 (s, 1H, OH). ^13 ^C-NMR (DMSO-d_6_, 75 MHz): 157.75, 142.14, 126.18, 123.65, 116.60. The compounds **1 b-1e** were obtained according to the above method using the other hydrazides.

#### 4.1.2. Synthesis of compounds 2a-2h

Taking compound **2a** as an example: compound **1a** (0.50 g, 3.1 mmol) and KOH (0.35 g, 6.2 mmol) were put into a flask with 5 mL of DMSO. The mixture was stirred for 5 min at 20 °C. Then added 1-bromo-3-chloropropane (0.98 g, 6.2 mmol) into the mixture and continued the reaction. After completion of the reaction indicated by the TLC (developing agent ratio: CH_2_Cl_2_/CH_3_OH = 15/1), the mixture was poured into 30 mL of water. The solution was extracted with dichloromethane three times. The organic layers were combined, washed with saturated salt water, dried over MgSO_4_, filtered, and concentrated. Purification by column chromatography (silica gel, 0-5% methanol in CH_2_Cl_2_) gave the compound **2a**. Chemical formula: C_11_H_12_ClN_3_O (MW = 237.69). m.p. 102–104 °C, yield 81%. ^1^H-NMR (300 MHz, DMSO-d_6_): *δ* 2.15–2.23 (m, 2H, OCH_2_CH_2_), 3.80 (t, 2H, *J* = 6.5 Hz, ClCH_2_), 4.16 (t, 2H, *J* = 6.1 Hz, OCH_2_), 7.13 (d, 2H, *J* = 9.0 Hz, Ph-H), 7.60 (d, 2H, *J* = 9.0 Hz, Ph-H), 9.00 (s, 2H, N=CH). ^13 ^C-NMR (75 MHz, DMSO-d_6_): *δ* 158.40, 142.03, 127.75, 123.40, 116.03, 65.22, 42.35, 32.05. As mentioned above, replaced the reactant **1a** by the alternative 4-(3-substituted-4*H*-1,2,4-triazol-4-yl)phenols (**1b-1e**) to give the compounds **2b-2e**. Compounds **2f**, **2g**, **2h** were obtained by the same method as above just replacing 1-bromo-3-chloropropane by 1-bromo-2-chloroethane, 1-bromo-4-chlorobutane, 1-bromo-2-chloropentane, respectively.

#### 4.1.3. Synthesis of compounds 3a-3q

Taking compound **3a** as an example: in a 100 mL round-bottom flask with 15 mL of acetonitrile, compound **2a** (0.40 g, 1.68 mmol), diethylamine (0.245 g, 3.36 mmol), K_2_CO_3_ (0.46 g, 3.36 mmol) and potassium iodide (0.56 g, 3.36 mmol) were added one by one. The mixture was heated up to 110 °C for 12–16 h. After cooing the mixture to 40 °C, it was filtered and dried by vacuum to obtain a residue. Purification by column chromatography (silica gel, 0–20% methanol in CH_2_Cl_2_) gave the compound **3a**. The same conditions were used to prepare the compounds **3b-3q**. Compounds **3a**, **3c**, **3d**, **3e**, **3 g**, **3k**, **3o**, and **3p** obtained as oils were transformed into the corresponding hydrochloride by hydrogen chloride in CH_2_Cl_2_.

#### 4.1.4. Characterisation for the target compounds

##### 4.1.4.1. 3-(4-(4H-1,2,4-triazol-4-yl)phenoxy)-N,N-diethylpropan-1-amine hydrochloride (3a)

Chemical formula: C_15_H_22_N_4_O × HCl (MW = 310.83). m.p. 105–106 °C, yield 82%. ^1^H-NMR (300 MHz, DMSO-d_6_): *δ* 1.25 (t, 6H, *J* = 7.2 Hz, CH_3_), 2.18 (t, 2H, *J* = 9.0 Hz, OCH_2_CH_2_), 3.08–3.16 (m, 6H, N(CH_2_)_3_), 4.17 (t, 2H, *J* = 6.0 Hz, OCH_2_), 7.19 (d, 2H, *J* = 8.8 Hz, Ph-H), 7.75 (d, 2H, *J* = 8.8 Hz, Ph-H), 9.84 (s, 2H, N=CH), 10.96 (s, 1H, HCl). ^13 ^C-NMR (75 MHz, DMSO-d_6_): *δ* 159.34, 142.39, 126.44, 124.35, 116.08, 66.00, 48.04, 46.59, 23.40, 8.87. ESI-HRMS calculated for C_15_H_23_N_4_O^+^ ([M-Cl]^+^): 275.1866; found: 275.1860.

##### 4.1.4.2. 3-(4-(4H-1,2,4-triazol-4-yl)phenoxy)-N,N-dipropylpropan-1-amine hydrochloride (3b)

Chemical formula: C_17_H_26_N_4_O × HCl (MW = 338.88). m.p. 180–183 °C, yield 62%. ^1^H-NMR (300 MHz, CDCl_3_,): *δ* 1.05 (t, 6H, *J* = 7.3 Hz, CH_3_), 1.77–1.90 (m, 4H, N(CH_2_CH_2_)_2_), 2.27–2.37 (m, 2H, OCH_2_CH_2_), 3.15 (t, 4H, *J* = 8.0 Hz, N(CH_2_)_2_), 3.40 (t, 2H, *J* = 8.3 Hz, NCH_2_), 4.18 (t, 2H, *J* = 5.6 Hz, OCH_2_), 7.05 (d, 2H, *J* = 8.9 Hz, Ph-H), 7.43 (d, 2H, *J* = 8.9 Hz, Ph-H), 8.62 (s, 2H, CH=N)., 9.51 (s, 1H, HCl). ^13 ^C-NMR (75 MHz, CDCl_3_): *δ* 163.08, 146.48, 132.18, 128.41, 120.58, 69.93, 59.42, 55.13, 28.54, 21.98, 15.92. ESI-HRMS calculated for C_17_H_27_N_4_O^+^ ([M-Cl]^+^): 303.2179; found: 303.2178.

##### 4.1.4.3. 4-(4-(3-(pyrrolidin-1-yl)propoxy)phenyl)-4H-1,2,4-triazole hydrochloride (3c)

Chemical formula: C_15_H_20_N_4_O × HCl (MW = 308.81). m.p. 120–122 °C, yield 56%. ^1^H-NMR (300 MHz, DMSO-d_6_): *δ* 1.92–2.00 (m, 4H, NCH_2_CH_2_), 2.19–2.22 (m, 2H, OCH_2_CH_2_), 3.00–3.53 (m, 6H, N(CH_2_)_3_), 4.17 (t, 2H, *J* = 5.7 Hz, OCH_2_), 7.17 (d, 2H, *J* = 8.7 Hz, Ph-H), 7.72 (d, 2H, *J* = 8.7 Hz, Ph-H), 9.75 (s, 2H, N=CH), 11.45 (s, 1H, HCl). ^13 ^C-NMR (75 MHz, DMSO-d_6_): *δ* 159.25, 142.36, 126.57, 124.25, 116.07, 66.03, 53.21, 51.46, 25.53, 23.24. ESI-HRMS calculated for C_15_H_21_ClN_4_O^+^ ([M-Cl]^+^): 273.1710; found: 273.1711.

##### 4.1.4.4. 1-(3-(4-(4H-1,2,4-triazol-4-yl)phenoxy)propyl)piperazine dihydrochloride (3d)

Chemical formula: C_15_H_21_N_5_O × 2HCl (MW = 323.83). m.p. 146–148 °C, yield 52%. ^1^H-NMR (300 MHz, CDCl_3_): *δ* 2.20–2.30 (m, 2H, *J* = 7.0 Hz, OCH_2_CH_2_), 3.32 (t, 2H, *J* = 7.8 Hz, NCH_2_), 3.37–3.57 (m, 8H, Piperazine*-*H), 4.17 (t, 2H, *J* = 5.8 Hz, OCH_2_), 7.16 (d, 2H, *J* = 8.9 Hz, Ph-H), 7.68 (d, 2H, *J* = 8.9 Hz, Ph-H), 9.44 (s, 2H, CH=N), 10.04 (s, 2H, HCl). ^13 ^C-NMR (75 MHz, DMSO-d_6_): *δ* 158.91, 142.29, 127.01, 123.96, 116.10, 65.89, 53.41, 48.17, 45.78, 23.50. ESI-HRMS calculated for C_15_H_22_N_5_O^+^ ([M-2HCl + H]^+^): 288.1819; found: 288.1820.

##### 4.1.4.5. 1-(3-(4-(4H-1,2,4-triazol-4-yl)phenoxy)propyl)-4-methylpiperazine hydrochloride (3e)

Chemical formula: C_16_H_23_N_5_O × HCl (MW = 337.85). m.p. 220–223 °C, yield 65%. ^1^H-NMR (300 MHz, CDCl_3_): *δ* 2.26 (s, 2H, OCH_2_CH_2_), 2.84 (s, 3H, CH_3_), 3.35-3.66 (m, 10H, N(CH_2_)_5_), 4.17 (t, 2H, *J* = 5.2 Hz, OCH_2_), 7.16 (d, 2H, *J* = 8.7 Hz, Ph-H), 7.68 (d, 2H, *J* = 8.7 Hz, Ph-H), 9.46 (s, 2H, CH=N), 10.57 (s, 1H, HCl). ^13 ^C-NMR (75 MHz, DMSO-d_6_): *δ* 158.93, 142.30, 126.96, 123.99, 116.10, 65.90, 54.53, 48.75, 45.75, 23.60, 15.53. ESI-HRMS calculated for C_16_H_24_N_5_O^+^ ([M-Cl]^+^): 302.1975; found: 302.1976.

##### 4.1.4.6. 1-(3-(4-(4H-1,2,4-triazol-4-yl)phenoxy)propyl)-4-phenylpiperazine (3f)

Chemical formula: C_21_H_25_N_5_O (MW = 363.47). m.p. 172–174 °C, yield 64%. ^1^H-NMR (300 MHz, DMSO-d_6_): *δ* 1.91–1.97 (m, 2H, OCH_2_CH_2_), 2.47–2.55 (m, 6H, N(CH_2_)_3_), 3.12–3.15 (m, 4H, N(CH_2_)_2_), 4.09 (t, 2H, *J* = 6.2 Hz, OCH_2_), 6.76 (t, 1H, *J* = 7.2 Hz, Ph-H), 6.92 (d, 2H, *J* = 8.0 Hz, Ph-H), 7.10 (d, 2H, *J* = 8.9 Hz, Ph-H), 7.2 (t, 2H, *J* = 7.9 Hz, Ph-H), 7.58 (d, 2H, *J* = 8.9 Hz, Ph-H), 9.00 (s, 2H, CH=N). ^13 ^C-NMR (75 MHz, DMSO-d_6_): *δ* 158.70, 151.50, 142.03, 129.36, 127.52, 123.37, 119.21, 116.00, 115.77, 66.81, 54.82, 53.26, 48.68, 26.60. ESI-HRMS calculated for C_21_H_26_N_5_O^+^ ([M + H]^+^): 364.2132; found: 364.2133.

##### 4.1.4.7. 4-(3-(4-(4H-1,2,4-triazol-4-yl)phenoxy)propyl)morpholine hydrochloride (3g)

Chemical formula: C_15_H_20_N_4_O_2_ × HCl (MW = 324.81). m.p. 237–239 °C, yield 51%. ^1^H-NMR (300 MHz, CDCl_3_): *δ* 2.27 (q, 2H, *J* = 6.0 Hz, OCH_2_CH_2_), 3.12-3.48 (m, 6H, N(CH_2_)_3_), 3.92 (t, 4H, *J* = 7.7 Hz, OCH_2_), 4.16 (t, 2H, *J* = 6.0 Hz, OCH_2_), 7.14 (d, 2H, *J* = 8.9 Hz, Ph-H), 7.67 (d, 2H, *J* = 8.9 Hz, Ph-H), 9.52 (s, 2H, N=CH), 11.44 (s, 1H, HCl). ^13 ^C-NMR (75 MHz, DMSO-d_6_): *δ* 159.02, 142.22, 126.81, 123.93, 116.01, 65.94, 63.63, 53.95, 51.55, 23.37. ESI-HRMS calculated for C_15_H_21_N_4_O_2_^+^ ([M - Cl]^+^): 289.1659; found: 289.1658.

##### 4.1.4.8. 1–(3-(4-(4H-1,2,4-triazol-4-yl)phenoxy)propyl)piperidine (3h)

Chemical formula: C_16_H_22_N_4_O (MW = 286.38). m.p. 249–252 °C, yield 67%. ^1^H-NMR (300 MHz, DMSO-d_6_): *δ* 1.38–1.91 (m, 6H, NCH_2_CH_2_(CH_2_)_2_), 2.20–2.30 (m, 2H, OCH_2_CH_2_), 2.83–3.44 (m, 6H, N(CH_2_)_3_), 4.15 (t, 2H, *J* = 6.0 Hz, OCH_2_), 7.17 (d, 2H, *J* = 8.9 Hz, Ph-H), 7.70 (d, 2H, *J* = 8.9 Hz, Ph-H), 9.62 (s, 2H, CH=N). ^13 ^C-NMR (75 MHz, DMSO-d_6_): *δ* 159.09, 142.30, 126.78, 124.09, 116.07, 66.20, 53.67, 52.40, 23.59, 22.71, 21.90. ESI-HRMS calculated for C_16_H_23_N_4_O^+^ ([M + H]^+^): 287.1866; found: 287.1867.

##### 4.1.4.9. 1-(3-(4-(4H-1,2,4-triazol-4-yl)phenoxy)propyl)-4-phenylpiperidine (3i)

Chemical formula: C_22_H_26_N_4_O (MW = 362.48). m.p. 124–126 °C, yield 50%. ^1^H-NMR (300 MHz, DMSO-d_6_): *δ* 1.88–2.10 (m, 6H, NCH_2_CH_2_), 2.17–2.22 (m, 2H, NCH_2_), 2.56 (t, 1H, *J* = 6.0 Hz, NCH_2_CH_2_CH), 2.64 (t, 2H, *J* = 7.0 Hz, NCH_2_), 3.14 (t, 2H, *J* = 6.4 Hz, NCH_2_), 4.11 (t, 2H, *J* = 6.0 Hz, OCH_2_), 7.03-7.32 (m, 9H, Ph-H), 8.47 (s, 2H, CH=N). ^13 ^C-NMR (75 MHz, DMSO-d_6_): *δ* 159.49, 146.04, 141.92, 128.46, 126.83, 126.71, 126.24, 123.98, 115.85, 66.88, 55.36, 54.42, 42.51, 33.23, 26.68. ESI-HRMS calculated for C_22_H_27_N_4_O^+^ ([M + H]^+^): 363.2179; found: 363.2178.

##### 4.1.4.10. 1-(3-(4-(4H-1,2,4-triazol-4-yl)phenoxy)propyl)piperidine-4-carboxamide (3j)

Chemical formula: C_17_H_23_N_5_O_2_ (MW = 329.40). m.p. 196–198 °C, yield 68%. ^1^H-NMR (300 MHz, CDCl_3_): *δ* 1.74–2.06 (m, 7H, NCH_2_(CH_2_)_3_CH), 2.14–3.00 (m, 6H, N(CH_2_)_3_), 4.07 (d, 2H, *J* = 6.5 Hz, OCH_2_), 5.70 (s, 2H, NH_2_), 7.02 (d, 2H, *J* = 8.9 Hz, Ph-H), 7.28 (d, 2H, *J* = 8.9 Hz, Ph-H), 8.39 (s, 2H, CH=N). ^13 ^C-NMR (75 MHz, CDCl_3_): *δ* 177.53, 159.53, 141.87,126.30, 123.98, 115.81, 66.76, 54.96, 53.23, 42.71, 28.94, 26.71. ESI-HRMS calculated for C_17_H_24_N_5_O_2_^+^ ([M + H]^+^): 330.1925; found: 330.1926.

##### 4.1.4.11. 1-(3-(4–(3-methyl-4H-1,2,4-triazol-4-yl)phenoxy)propyl)piperidine hydrochloride (3k)

Chemical formula: C_17_H_24_ClN_4_O × HCl (MW = 336.86). m.p. 240–242 °C, yield 61%. ^1^H-NMR (300 MHz, CDCl_3_ + DMSO-d_6_): *δ* 1.86–2.43 (m, 8H, NCH_2_CH_2_CH_2_), 2.64 (s, 3H, N=CCH_3_), 2.94–3.55 (m, 6H, N(CH_2_)_3_), 4.22 (s, 2H, OCH_2_), 7.13 (d, 2H, *J* = 5.8 Hz, Ph-H), 7.55 (d, 2H, *J* = 5.8 Hz, Ph-H), 9.33 (s, 1H, CH=N), 11.31 (s, 1H, HCl). ^13 ^C- NMR (75 MHz, CDCl_3_ + DMSO-d_6_): *δ* 164.95, 156.81, 149.03, 132.21, 129.15, 120.75, 70.66, 59.08, 57.74, 28.43, 27.48, 26.65, 15.04. ESI-HRMS calculated for C_17_H_25_N_4_O^+^ ([M-Cl]^+^): 301.2023; found: 301.2025.

##### 4.1.4.12. 1-(3-(4-(3-phenyl-4H-1,2,4-triazol-4-yl)phenoxy)propyl)piperidine (3l)

Chemical formula: C_22_H_26_N_4_O (MW = 362.48). m.p. 88–90 °C, yield 59%. ^1^H-NMR (300 MHz, DMSO-d_6_): *δ* 1.68 (s, 2H, NCH_2_CH_2_CH_2_), 1.84–1.92 (m, 4H, NCH_2_(CH_2_)_2_, 2.18–2.30 (m, 2H, OCH_2_CH_2_), 3.18-3.25 (m, 6H, N(CH_2_)_3_), 4.13 (t, 2H, *J* = 6.0 Hz, OCH_2_), 7.00 (d, 2H, *J* = 8.9 Hz, Ph-H), 7.23 (d, 2H, *J* = 8.9 Hz, Ph-H), 7.32-7.46 (m, 5H, Ph-H), 8.45 (s, 1H, CH=N). ^13 ^C-NMR (75 MHz, CDCl_3_): *δ* 163.64, 157.72, 150.07, 134.62, 133.40, 133.26, 132.35, 132.05, 131.39, 120.31, 70.19, 59.33, 58.11, 28.99, 28.09, 26.66. ESI-HRMS calculated for C_22_H_27_N_4_O^+^ ([M + H]^+^): 363.2179; found: 363.2178.

##### 4.1.4.13. 1-(3-(4-(3-(4-chlorophenyl)-4H-1,2,4-triazol-4-yl)phenoxy)propyl)piperidine (3m)

Chemical formula: C_22_H_25_ClN_4_O (MW = 396.92). m.p. 165–167 °C, yield 75%. ^1^H-NMR (300 MHz, CDCl_3_): *δ* 1.54 (s, 2H, NCH_2_CH_2_CH_2_), 1.72 (s, 4H, NCH_2_(CH_2_)_2_), 2.09–2.14 (m, 2H, OCH_2_CH_2_), 3.08–3.33 (m, 6H, N(CH_2_)_3_), 4.10 (t, 2H, *J* = 6.0 Hz, OCH_2_), 7.06 (d, 2H, *J* = 8.8 Hz, Ph-H), 7.34 (d, 2H, *J* = 8.8 Hz, Ph-H), 7.41 (d, 2H, *J* = 8.6 Hz, Ph-H), 7.48 (d, 2H, *J* = 8.6 Hz, Ph-H), 8.79 (s, 1H, CH=N). ^13 ^C-NMR (75 MHz, DMSO-d_6_): *δ* 159.14, 151.96, 146.43, 135.01, 130.54, 129.21, 128.04, 127.56, 126.12, 115.85, 65.98, 54.08, 52.99, 29.45, 24.31, 23.56. ESI-HRMS calculated for C_22_H_26_ClN_4_O^+^ ([M + H]^+^): 397.1790; found: 397.1791.

##### 4.1.4.14. 1-(3-(4-(3-([1,1’-biphenyl]-4-yl)-4H-1,2,4-triazol-4-yl)phenoxy)propyl)piperidine (3n)

Chemical formula: C_28_H_30_N_4_O (MW = 438.58). m.p. 66–68 °C, yield 58%. ^1^H-NMR (300 MHz, DMSO-d_6_): *δ* 1.73 (s, 2H, NCH_2_CH_2_CH_2_), 2.01–2.09 (m, 4H, NCH_2_CH_2_), 2.39–2.48 (m, 2H, OCH_2_CH_2_), 3.12–3.20 (m, 6H, N(CH_2_)_3_), 4.14 (t, 2H, *J* = 6.0 Hz, OCH_2_), 6.96 (d, 2H, *J* = 8.8 Hz, Ph-H), 7.20 (d, 2H, *J* = 8.8 Hz, Ph-H), 7.35-7.58 (m, 9H, Ph-H), 8.34 (s, 1H, CH=N). ^13 ^C-NMR (75 MHz, DMSO-d_6_): *δ* 158.97, 153.04, 145.16, 142.55, 139.81, 128.89, 128.86, 127.91, 127.63, 127.63, 127.22, 127.01, 125.05, 115.61, 65.70, 55.17, 53.69, 24.32, 23.08, 22.30. ESI-HRMS calculated for C_28_H_31_N_4_O^+^ ([M + H]^+^): 439.2492; found: 439.2494.

##### 4.1.4.15. 1-(2-(4–(4H-1,2,4-triazol-4-yl)phenoxy)ethyl)piperidine hydrochloride (3o)

Chemical formula: C_15_H_20_N_4_O × HCl (MW = 308.81). m.p. 85–87 °C, yield 72%. ^1^H-NMR (300 MHz, CDCl_3_): *δ* 1.63–1.96 (m, 6H, NCH_2_(CH_2_)_2_CH_2_), 2.95–3.07 (m, 2H, OCH_2_CH_2_), 3.48 (t, 4H, *J* = 5.5 Hz, N(CH_2_)_2_), 4.55 (t, 2H, *J* = 5.1 Hz, OCH_2_), 7.23 (d, 2H, *J* = 8.8 Hz, Ph-H), 7.75 (d, 2H, *J* = 8.8 Hz, Ph-H), 9.69 (s, 2H, CH=N), 11.21 (s, 1H, HCl). ^13 ^C-NMR (75 MHz, CDCl_3_): *δ* 158.38, 142.33, 127.14, 124.20, 116.33, 63.37, 54.89, 53.01, 22.70, 21.68. ESI-HRMS calculated for C_15_H_21_N_4_O^+^ ([M-Cl]^+^): 273.1710; 273.1711.

##### 4.1.4.16. 1-(4–(4-(4H-1,2,4-triazol-4-yl)phenoxy)butyl)piperidine hydrochloride (3p)

Chemical formula: C_17_H_24_N_4_O × HCl (MW = 336.86). m.p. 80–82 °C, yield 69%. ^1^H-NMR (300 MHz, CDCl_3_): *δ* 1.88–2.21 (m, 8H, NCH_2_(CH_2_)_3_CH_2_), 2.22–2.36 (m, 2H, OCH_2_CH_2_), 2.82–3.73 (m, 6H, N(CH_2_)_3_), 4.08 (t, 2H, *J* = 5.8 Hz, OCH_2_), 7.00 (d, 2H, *J* = 8.9 Hz, Ph-H), 7.30 (d, 2H, *J* = 8.9 Hz, Ph-H), 8.49 (s, 2H, CH=N). 10.22 (s, 1H, HCl). ^13 ^C-NMR (75 MHz, CDCl_3_): *δ* 158.93, 141.86, 126.87, 123.86, 115.89, 67.27, 56.93, 53.26, 26.37, 22.50, 21.88, 20.76. ESI-HRMS calculated for C_17_H_25_N_4_O^+^ ([M-Cl]^+^): 301.2023; found: 301.2021.

##### 4.1.4.17. 1-(5-(4-(4H-1,2,4-triazol-4-yl)phenoxy)pentyl)piperidine (3q)

Chemical formula: C_18_H_26_N_4_O × HCl (MW = 314.43). m.p. 70–74 °C, yield 77%. ^1^H-NMR (300 MHz, CDCl_3_): *δ* 1.56–2.09 (m, 14H, NCH_2_(CH_2_)_3_(CH_2_)_2_CH_2_), 3.04–3.09 (m, 4H, N(CH_2_)_2_), 4.01 (t, 2H, *J* = 6.0 Hz, OCH_2_), 7.00 (d, 2H, *J* = 8.8 Hz, Ph-H), 7.30 (d, 2H, *J* = 8.8 Hz, Ph-H), 8.44 (s, 2H, CH=N). ^13 ^C-NMR (75 MHz, CDCl_3_): *δ* 159.25, 141.88, 126.69, 123.91, 115.86, 67.76, 57.25, 53.28, 28.32, 23.37, 22.49, 21.92, 21.88. ESI-HRMS calculated for C_18_H_27_N_4_O^+^ ([M + H]^+^): 315.2179; found: 315.2180.

### 4.2. Pharmacology

#### 4.2.1. In vitro screening

##### 4.2.1.1. Cell culture and transfection

Human thalamus poly-A RNA (Clontech, Palo Alto, CA, USA) was used to clone the hH_3_R gene by RT-PCR. DNA PCR primers were designed in the light of the reported human histamine receptor gene sequences (GenBank accession no.AF140538). HEK-293 cells were cultured and transfected for the luciferase assay. The detailed procedures were described in the previous publication^40^.

##### 4.2.1.2. CRE-driven reporter gene assay

Stable HEK-293 cells, which had been co-transfected with H_3_R and pCRE-Luc, were seeded in a 96-well plate overnight, and were grown to 90–95% confluence^43^^,^^44^. Then the cells were treated with various concentrations of tested compounds in serum-free DMEM and incubated for 20 min. Cells were then stimulated with 100 nM Histamine in serum-free DMEM containing 2 µM Forskolin and incubated for 4 h at 37 °C. Firefly luciferase assay kits (Ken-real, Shanghai, China) were used to determine the luciferase activity.

#### 4.2.2. *In vivo* pharmacology

##### 4.2.2.1. Drugs and animals

Valproic acid (VPA) was obtained from melongpharma, Dalian, China. Ciproxifan maleate was purchased from Shanghai Hanxiang Biotechnology Co., Ltd, China. R-(α)-methyl-histamine (RAMH), Pitolisant (PIT), and Pentylenetetrazol (PTZ) were bought from Macklin Co. KunMing mice were purchased from Changsha tianqin Biotechnology Co., Ltd, China and used with the body weight 20–25 g. Procedures involving animals were performed according to the Guide for the Care and Use of Laboratory Animals (8th Edition, National Academies Press, Washington, DC), and was approved by the local animal ethics committee (Institutional Animal Ethics Committee of Jinggangshan University, approval number: 201906018).

##### 4.2.2.2. MES-induced seizure

Ear stimulation with alternating current (0.2 s, 60 Hz, 50 mA) was used to induce the seizures in mice. The reduction or abolition of the hind limb tonic extension (THLE) of mice was considered protective against the MES-induced seizures^29^^,^^48^. Test compounds and positive drugs VPA (300 mg/kg) and PIT (10 mg/kg) were *i.p.* administrated half an hour prior to the electric stimulation. To investigate the mechanism of action, the most promising one **3m** was chose for a further test. In one group of animals of seven mice, compound **3m** (10 mg/kg) co-injected with RAMH 10 mg/kg with 5 min interval. The animals in other three groups were single treated with RAMH 10 mg/kg, compound **3m** 10 mg/kg, and vehicle, respectively.

##### 4.2.2.3. PTZ-induced seizures

PTZ (85 mg/kg) was injected subcutaneously to induce seizures. Firstly, vehicle, tested compounds **3a-3q** (10 mg/kg), and positive controls (VPA 300 mg/kg, PIT 10 mg/kg) were administered *i.p.* After 30 min, PTZ was injected to all the animals. Animals were observed for 30 min (experiment period) for any convulsion signs, and graded scores were used to assess the seizures severity. The following are the specific meaning of graded scores: score 0 = normal, score 1 = eyelids or facial twitches, score 2 = agitation with body twisting, score 3 = myo-clonic jerks or rearing, score 4 = turn over into one side position or running violently, and score 5 = turn over into back position, limbs tonic extension, or die during the experiment period^30^.

#### 4.2.3. Statistics

GraphPad Prism was used for statistical analysis. All data *in vivo* were presented as the mean ± standard error of mean (SEM). One-way analysis of variance (ANOVA), followed by the Dunnett’s post-test were conducted for multiple comparisons. The statistical significance was defined as *p* values <0.05.

#### 4.2.4. Docking

##### 4.2.4.1. Homology modelling

Crystal structure of the histamine H_1_ receptor (PDB ID: 3RZE) was used to construct the H_3_ receptor homology model^45^. The hH_3_R primary sequence was downloaded from the Universal Protein Resource (UniProt ID: Q9Y5N1). DS MODELLER (Discovery Studio 2019) was used to construct a 3D model of the H_3_R. Then the model was assessed in accordance with the PDF Total Energy and the Profile-3D procedure. The 3D model of H_3_R with the lowest PDF total energy was selected for the next docking test.

##### 4.2.4.2. Molecular docking

To consider the conformation of the protein and its ligands, Flexible Docking (Discovery Studio 2019) was used for the docking procedure. The initial ligand and water were removed, and hydrogen atoms were added. Three-dimensional structures of compounds **3h, 3m** as well as PIT were generated and then placed into the protein structure during the molecular docking procedure. Interactions of the protein with **3h, 3m**, and PIT were analysed.

## References

[1] Beghi E. Addressing the burden of epilepsy: many unmet needs. Pharmacol Res 2016;107:79–84. 26952026 10.1016/j.phrs.2016.03.003

[2] Singh SP, Ankaraneni RS, Antony AR. Evidence-based guidelines for the management of epilepsy. Neurol India 2017;65:S6–S11. 28281490 10.4103/neuroindia.NI_1027_16

[3] Gaitatzis A, Sander JW. The long-term safety of antiepileptic drugs. CNS Drugs 2013;27:435–55. 23673774 10.1007/s40263-013-0063-0

[4] Błaszczyk B, Lasón W, Czuczwar S. Antiepileptic drugs and adverse skin reactions: an update. Pharmacol Rep 2015;67:426–34. 25933949 10.1016/j.pharep.2014.11.009

[5] Ferrer P, Ballarín E, Sabaté M, et al. Antiepileptic drugs and suicide: a systematic review of adverse effects. Neuroepidemiology 2014;42:107–20. 24401764 10.1159/000356807

[6] Kiviranta T, Tuomisto L, Airaksinen EM. Histamine in cerebrospinal fluid of children with febrile convulsions. Epilepsia 1995;36:276–80. 7614912 10.1111/j.1528-1157.1995.tb00996.x

[7] Zhang LS, Chen Z, Huang YW, et al. Effects of endogenous histamine on seizure development of pentylenetetrazole-induced kindling in rats. Pharmacology 2003;69:27–32. 12886027 10.1159/000071263

[8] Kamei C, Ishizawa K, Kakinoki H, Fukunaga M. Histaminergic mechanisms in amygdaloid-kindled seizures in rats. Epilepsy Res 1998;30:187–94. 9657646 10.1016/s0920-1211(98)00005-9

[9] Yokoyama H. The role of central histaminergic neuron system as an anticonvulsive mechanism in developing brain. Brain Dev 2001;23:542–7. 11701252 10.1016/s0387-7604(01)00261-3

[10] Chen Z, Li WD, Zhu LJ, et al. Effects of histidine, a precursor of histamine, on pentylenetetrazole-induced seizures in rats. Acta Pharmacol Sin 2002;23:361–6. 11931695

[11] Arrang JM, Garbarg M, Schwartz JC. Auto-inhibition of brain histamine release mediated by a novel class (H3) of histamine receptor. Nature 1983;302:832–7. 6188956 10.1038/302832a0

[12] Brown RE, Stevens DR, Haas HL. The physiology of brain histamine. Prog Neurobiol 2001;63:637–72. 11164999 10.1016/s0301-0082(00)00039-3

[13] Lu CW, Lin TY, Chang CY, et al. Ciproxifan, a histamine H3 receptor antagonist and inverse agonist, presynaptically inhibits glutamate release in rat hippocampus. Toxicol Appl Pharmacol 2017;319:12–21. 28132918 10.1016/j.taap.2017.01.017

[14] Bhowmik M, Khanam R, Vohora D. Histamine H3 receptor antagonists in relation to epilepsy and neurodegeneration: a systemic consideration of recent progress and perspectives. Br J Pharmacol 2012;167:1398–414. 22758607 10.1111/j.1476-5381.2012.02093.x PMC3514756

[15] Kakinoki H, Ishizawa K, Fukunaga M, et al. The effects of histamine H3-receptor antagonists on amygdaloid kindled seizures in rats. Brain Res Bull 1998;46:461–5. 9739010 10.1016/s0361-9230(98)00048-3

[16] Vohora D, Pal SN, Pillai KK. Thioperamide, a selective histamine H3 receptor antagonist, protects against PTZ-induced seizures in mice. Life Sci 2000;66:PL297–301. 10834305 10.1016/s0024-3205(00)00548-8

[17] Ishizawa K, Chen Z, Okuma C, et al. Participation of GABAergic and histaminergic systems in inhibiting amygdaloid kindled seizures. Jpn J Pharmacol 2000;82:48–53. 10874588 10.1254/jjp.82.48

[18] Vohora D, Pal SN, Pillai KK. Histamine and selective H3-receptor ligands: a possible role in the mechanism and management of epilepsy. Pharmacol Biochem Behav 2001;68:735–41. 11526971 10.1016/s0091-3057(01)00474-9

[19] Zhang LS, Chen Z, Ren K, et al. Effects of clobenpropit on pentylenetetrazole-kindled seizures in rats. Eur J Pharmacol 2003;482:169–75. 14660019 10.1016/j.ejphar.2003.09.066

[20] Yokoyama H, Onodera K, Maeyama K, et al. Clobenpropit (VUF-9153), a new histamine H3 receptor antagonist, inhibits electrically induced convulsions in mice. Eur J Pharmacol 1994;260:23–8. 7957622 10.1016/0014-2999(94)90005-1

[21] Hirai T, Okuma C, Harada C, et al. Development of amygdaloid kindling in histidine decarboxylase-deficient and histamine H1 receptor-deficient mice. Epilepsia 2004;45:309–13. 15030492 10.1111/j.0013-9580.2004.19303.x

[22] Uma Devi P, Manocha A, Khanam R, Vohora D. Beneficial interaction between clobenpropit and pyridoxine in prevention of electroshock-induced seizures in mice: lack of histaminergic mechanisms. Hum Exp Toxicol 2011;30:84–8. 20511290 10.1177/0960327110372398

[23] Yokoyama H, Onodera K, Iinuma K, Watanabe T. Effect of thioperamide, a histamine H3 receptor antagonist, on electrically induced convulsions in mice. Eur J Pharmacol 1993;234:129–33. 8386092 10.1016/0014-2999(93)90717-v

[24] Sadek B, Saad A, Subramanian D, et al. Anticonvulsant and procognitive properties of the non-imidazole histamine H3 receptor antagonist DL77 in male adult rats. Neuropharmacology 2016;106:46–55. 26525191 10.1016/j.neuropharm.2015.10.023

[25] Kuder KJ, Łażewska D, Kaleta M, et al. Synthesis and biological activity of novel tert-amylphenoxyalkyl (homo)piperidine derivatives as histamine H3R ligands. Bioorg Med Chem 2017;25:2701–12. 28372935 10.1016/j.bmc.2017.03.031

[26] Alachkar A, Łażewska D, Latacz G, et al. Studies on anticonvulsant effects of novel histamine H_3_R antagonists in electrically and chemically induced seizures in rats. Int J Mol Sci 2018;19:3386. 10.3390/ijms19113386 PMC6274786 30380674

[27] Szczepańska K, Karcz T, Mogilski S, et al. Synthesis and biological activity of novel tert-butyl and tert-pentylphenoxyalkyl piperazine derivatives as histamine H3R ligands. Eur J Med Chem 2018;152:223–34. 29723785 10.1016/j.ejmech.2018.04.043

[28] Łażewska D, Kaleta M, Hagenow S, et al. Novel naphthyloxy derivatives – potent histamine H3 receptor ligands. Synthesis and pharmacological evaluation. Bioorg Med Chem 2018;26:2573–85. 29681486 10.1016/j.bmc.2018.04.023

[29] Sadek B, Schwed JS, Subramanian D, et al. Non-imidazole histamine H_3_ receptor ligands incorporating antiepileptic moieties. Eur J Med Chem 2014;77:269–79. 24650714 10.1016/j.ejmech.2014.03.014

[30] Sadek B, Saad A, Schwed JS, et al. Anticonvulsant effects of isomeric nonimidazole histamine H3 receptor antagonists. Drug Des Devel Ther 2016;10:3633–51. 10.2147/DDDT.S114147 PMC5106240 27853355

[31] Schwartz J-C. The histamine H3 receptor: from discovery to clinical trials with pitolisant. British Journal of PharmacologyBr J Pharmacol 2011;163:713–21. 21615387 10.1111/j.1476-5381.2011.01286.x PMC3111674

[32] Kasteleijn-Nolst Trenité D, Parain D, Genton P, et al. Efficacy of the histamine 3 receptor (H3R) antagonist pitolisant (formerly known as tiprolisant; BF2.649) in epilepsy: dose-dependent effects in the human photosensitivity model. Epilepsy Behav 2013;28:66–70. 23665640 10.1016/j.yebeh.2013.03.018

[33] Deng XQ, Wei CX, Li FN, et al. Design and synthesis of 10-alkoxy-5, 6-dihydro-triazolo[4,3-d]benzo[f][1,4]oxazepine derivatives with anticonvulsant activity. Eur J Med Chem 2010;45:3080–6. 20416982 10.1016/j.ejmech.2010.03.041

[34] Cui XS, Chen J, Chai KY, Lee JS, et al. Synthesis and anticonvulsant evaluation of 3-substituted-4-(4-hexyloxyphenyl)-4H-1,2,4-triazoles. Med Chem Res 2009;18:49–58.

[35] Ayati A, Emami S, Foroumadi A. The importance of triazole scaffold in the development of anticonvulsant agents. Eur J Med Chem 2016;109:380–92. 26826582 10.1016/j.ejmech.2016.01.009

[36] Guan LP, Quan ZS. 3,4-DHQLO and triazole and its related analogues with anticonvulsant effects. Mini Rev Med Chem 2016;16:323–42. 25553427 10.2174/1389557515666150101100909

[37] Song MX, Deng XQ. Recent developments on triazole nucleus in anticonvulsant compounds: a review. J Enzyme Inhib Med Chem 2018;33:453–78. 29383949 10.1080/14756366.2017.1423068 PMC6010125

[38] Guan LP, Zhao DH, Jiang Z, et al. Evaluation of anticonvulsant activity of QUAN-0806 in various murine experimental seizure models. Pharmazie 2009;64:248–51. 19435143

[39] Deng XQ, Quan LN, Song MX, et al. Synthesis and anticonvulsant activity of 7-phenyl-6,7-dihydro-[1,2,4]triazolo[1,5-a]pyrimidin-5(4H)-ones and their derivatives. Eur J Med Chem 2011;46:2955–63. 21536355 10.1016/j.ejmech.2011.04.020

[40] Deng XD, Song MX, Wang SB, Quan ZS. Synthesis and evaluation of the anticonvulsant activity of 8-alkoxy-4,5-dihydrobenzo[b][1,2,4]triazolo[4,3-d][1,4]thiazepine derivatives. J Enzyme Inhib Med Chem 2014;29:272–80. 23477412 10.3109/14756366.2013.776555

[41] Kaproń B, Łuszczki J, Paneth A, et al. Molecular mechanism of action and safety of 5-(3-chlorophenyl)-4-hexyl-2,4-dihydro-3H-1,2,4-triazole-3-thione – a novel anticonvulsant drug candidate. Int J Med Sci 2017;14:741–9. 28824309 10.7150/ijms.20001 PMC5562128

[42] Kaproń B, Łuszczki JJ, Płazińska A, et al. Development of the 1,2,4-triazole-based anticonvulsant drug candidates acting on the voltage-gated sodium channels. Insights from in-vivo, in-vitro, and in-silico studies. Eur J Pharm Sci 2019;129:42–57. 30594731 10.1016/j.ejps.2018.12.018

[43] Huang W, Tang T, Shi Y, et al. Searching for the multi-target-directed ligands against Alzheimer's disease: discovery of quinoxaline-based hybrid compounds with AChE, H_3_R and BACE 1 inhibitory activities. Bioorg Med Chem 2011;19:7158–67. 22019465 10.1016/j.bmc.2011.09.061

[44] Shi Y, Sheng R, Zhong T, et al. Identification and characterization of ZEL-H16 as a novel agonist of the histamine H3 receptor. PLoS One 2012;7:e42185. 22870296 10.1371/journal.pone.0042185 PMC3411647

[45] Shimamura T, Shiroishi M, Weyand S, et al. Structure of the human histamine H1 receptor complex with doxepin. Nature 2011;475:65–70. 21697825 10.1038/nature10236 PMC3131495

[46] Kim S-K, Fristrup P, Abrol R, Goddard WA. Structure-based prediction of subtype selectivity of histamine H3 receptor selective antagonists in clinical trials. J Chem Inf Model 2011;51:3262–74. 22035233 10.1021/ci200435b PMC3246544

[47] Ghamari N, Zarei O, Reiner D, et al. Histamine H3 receptor ligands by hybrid virtual screening, docking, molecular dynamics simulations, and investigation of their biological effects. Chem Biol Drug Des 2019;93:832–43. 30586225 10.1111/cbdd.13471

[48] Song MX, Wang ZY, He SH, et al. Synthesis and evaluation of the anticonvulsant activities of 4-(2-(alkylthio)benzo[d]oxazol-5-yl)-2,4-dihydro-3H-1,2,4-triazol-3-ones. Molecules 2018;23:756. 10.3390/molecules23040756 PMC6017283 29587394

